# Rat models of reward deficits in psychiatric disorders

**DOI:** 10.1016/j.cobeha.2018.05.001

**Published:** 2018-08

**Authors:** Chloe L. Slaney, Claire A. Hales, Emma S.J. Robinson

**Affiliations:** School of Physiology, Pharmacology & Neuroscience, Biomedical Sciences Building, University Walk, Bristol BS8 4PX, UK

## Abstract

Loss of interest in rewarding activities is a hallmark of many psychiatric disorders and may be relevant for neurodegenerative disorders and patients suffering from brain injury. There is increasing evidence that deficits in reward-related behaviour are more complex than previously described. The traditional view of anhedonia as ‘the inability to experience pleasure’ may be too limited to fully encompass the types of reward deficit observed in these patients. Developments in methods to measure different aspects of reward processing in humans and animals are starting to provide insights into the complexity of this behaviour. In this article we consider the rodent models which have traditionally been used to study reward deficits in psychiatric disorders and consider their limitations relative to clinical findings. We then discuss work where methods derived from human neuropsychological tests are providing insights into the complexity of reward-related behaviour. Specifically, we consider tasks which investigate different aspects of reward-related behaviour focusing on learning and memory as well as decision-making and consider what these may mean in terms of how we model reward deficits in rodents.

**Current Opinion in** Behavioral **Sciences** 2018, **22**:136–142

This review comes from a themed issue on **Apathy and motivation**

Edited by **Christopher Pryce** and **Masud Husain**

For a complete overview see the Issue and the Editorial

Available online 12th June 2018

**https://doi.org/10.1016/j.cobeha.2018.05.001**

2352-1546/© 2018 The Authors. Published by Elsevier Ltd. This is an open access article under the CC BY license (http://creativecommons.org/licenses/by/4.0/).

## Introduction

Deficits in reward processing are observed across a range of psychiatric disorders [1, 2, 3, 4, 5, 6]. More broadly, impairments in reward processing may contribute to the observed motivational deficits, loss of interest in social interaction, and apathy. Whilst reward deficits are clearly an important feature of these clinical conditions, there are currently no treatments which specifically target these symptoms. Animal models are an important element of aetiological studies and drug development programmes. However, using animals to study complex human psychological symptoms is often challenging. In this article, we consider why traditional consummatory and motivational tests for anhedonia may be limited in terms of providing a valid translational approach to studying the reward deficits that are most prevalent in these different patient populations. We also consider whether new tasks, which look at more complex processing of reward information, may provide a better approach. Specifically, we discuss new data from models looking at reward learning and decision-making as well as studies where biases in reward-related behaviour have been linked to changes in affective state.

## What is anhedonia?

**Figure 1:**
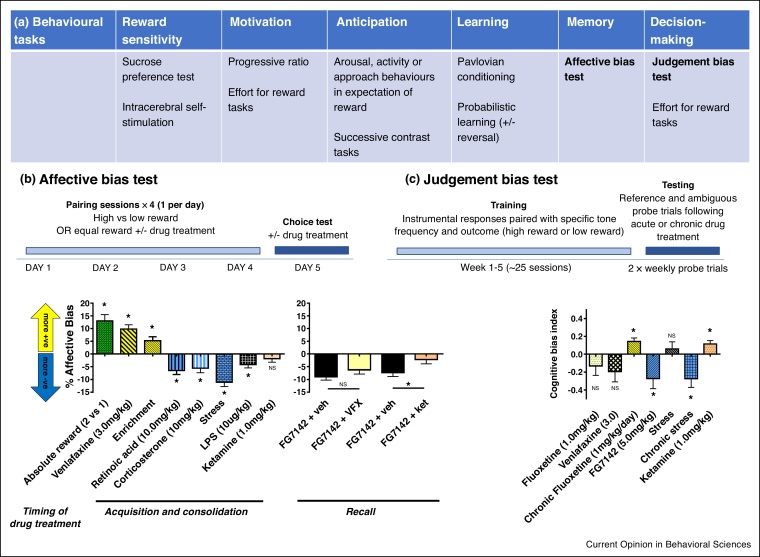
Rodent tasks which provide quantified measures of the different aspects of reward processing have been developed. Summarised in table **(a)** are examples of these different methods. Recent developments have seen a shift from the more typical measures of reward deficits based on changes in hedonia or motivation to methods which measure the cognitive aspects of reward such as learning memory and decision-making. Panels **(b,c)** illustrate two tasks which we have recently developed to study how reward-related behaviour is altered by affective state. The affective bias test is used to study biases in reward learning and memory and the judgement bias task which provides a method to study interpretation biases in the interpretation of ambiguous information and an animal’s anticipation of positive or negative outcomes. Panel (b) illustrates the ABT method and some of the data obtained following manipulations administered during the learning phase of the task only (acquisition and consolidation). We can also test how these biases are subsequently modulated by also administering treatments before the preference test (recall) to determine if they alter the previously learnt bias. These studies have revealed that antidepressant and pro-depressant manipulations induce a subsequent bias in the reward-related memory. Following induction of a negative bias, pre-treatment with ketamine but not the conventional antidepressant venlafaxine results in an attenuation of the bias suggesting these different types of antidepressant can differentially modulate reward-related learning and memory. Dissociable effects between conventional and rapid onset antidepressants have also been observed in the judgement bias task with evidence of difference in the time course of effects. As illustrated in panel (c), the judgement bias task involves first training animals in an operant task where responses to obtain different values of reward, (or reward versus punishment avoidance) are first trained. Animals’ interpretation biases are then probes using a testing phase where both reference and intermediate ambiguous cues are presented, and the animals responses recorded. In this measure of reward-related decision-making, chronic but not acute treatment with antidepressants induces a shift in decision-making biases. In contrast, ketamine induces an immediate positive bias suggesting different underlying mechanisms of action which may help explain the temporal differences observed with these treatments in patients. More details of the methods for the affective bias test and judgement bias task are discussed in the main text.

Historically, anhedonia was defined as an ‘inability to experience pleasure’ [7]. However, in the last 20 years, knowledge relating to the neurobiology of reward and how we consider this in relation to anhedonia has developed. This has resulted in a growing interest in how we define anhedonia and consequently how we model this in rodents. Whilst the exact definitions are debated (see Table 1 and review articles [8, 9, 10, 11,12^•^,^13^,^14^]), it is suggested that symptoms of anhedonia observed in patients may be due to deficits in one or several different components of reward processing: firstly, consummatory experience of reward, secondly motivation for reward, thirdly reward learning and finally decision making. In relation to major depressive disorder, impairments in reward-related behaviour are more broadly set out in the DSM-5 as a ‘loss of interest or pleasure in previously rewarding activities’. Reward-related behavioural deficits are also considered within the recent concept of Research Domains Criteria (RDoC) framework for mental health research [15, 16, 17]. The positive valance system makes up one of the key domains which has been included in this framework. In this article, we will focus our discussion on methods to assess these different subcomponents of reward (see Figure 1, panel a for summary). We briefly discuss why traditional consummatory and motivational tests for anhedonia may be limited in terms of providing a valid translational approach to studying the reward deficits that are most prevalent in different patient populations. We also discuss whether recently developed methods looking at reward learning, memory and decision-making may provide a better approach. Specifically, we discuss new data from behavioural tasks which have been looking at reward learning and decision-making as well as studies where biases in reward-related cognition have been linked to changes in the emotional state of the animal, more commonly referred to as affective state in non-human species.

**Table 1:** Summary of some of the key papers relating to the discussions about how we define anhedonia and reward-related deficits in psychiatric disorders [7,8,9,12^•^,^13^,^14^]

Year	Definition of anhedonia	Reference
1896	‘The inability to experience pleasure’	Ribot (1896)
2003	Liking Wanting	Robinson and Berridge (2003)
2008	Liking, wanting, learning (pleasure cycle = appetitive, consummatory and satiety	Berridge and Kringelbach (2008)
2011	Distinction between consummatory, motivational and decision-making	Treadway and Zald (2011)
2012	Distinction between anhedonia and cognitive aspects of reward	Der-Avakian and Markou (2012)
2015	`Impairments in the ability to pursue, experience and/or learn about pleasure’	Thomsen (2015)

## Limitations associated with consummatory and motivational deficits in reward-related behaviour in rats

Most studies investigating reward processing in rodents have used tasks based on consummatory behaviour (hedonia) and motivation for reward (see [1,2,12^•^,^13^,^14^,^18^,^19^] for detailed review of relevant methodology). For example, reward sensitivity can be measured directly using intracerebral self-stimulation methods where deficits resulting from chronic stress are observed as an increase in the stimulation threshold [20]. A simpler and more commonly used method to study anhedonia, particularly for depression research, has been the sucrose preference test (SPT) where the ability of an animal to detect and show a preference for a weak sucrose or saccharin solution over water is measured [21]. Animals in putative negative affective states following exposure to chronic stress show a reduced sucrose preference [21, 22, 23]. However, not all depression models show impairments in the SPT and studies in Schizophrenia models also fail to observe deficits [1,24]. Additionally, in the human literature, depressed patients do not exhibit deficits in a similar sweet taste test [25,26] suggesting limited translational validity of the SPT.

Motivation for reward tasks, such as progressive ratio and effort-based tasks, provide an alternative method to study reward-related behaviours [15,27]. In the progressive ratio task, over several trials rats are required to perform incrementally higher operant responses (e.g. press a lever) in order to obtain the same amount of reward. Motivation in this task is determined as the point at which rats stop responding (i.e. their ‘breakpoint’). Whilst this task displays reasonable translational validity, with both humans and rodents displaying motivational deficits related to dopamine depletion [28, 29, 30] models of depression (e.g. chronic mild stress and maternal separation) and other psychiatric disorders in rodents display less consistent changes in motivation [31,32^•^,^33^], although also see [34^•^]. In progressive ratio tasks, increased effort (number of responses) is also associated with an increase in time to obtain reward and therefore may be confounded by motor impairments. There is also the potential for animal’s tolerance to delayed reward to contribute to behavioural outcomes which, whilst potentially of interest, may relate more to neural circuits modulating impulse control as opposed to reward. Because of the limitations of the standard progressive ratio task, effort-based choice tasks have been developed where animals are required to choose between an easy to obtain low-value reward versus a high-value/high-effort reward [5,35^••^,^36^,37^•^,^38^,^39^]. This task requires the animal to make a choice based on motivation for the different reward option and hence also models decision-making behaviour. Validation of the model is still limited but there is a clear translational advantage with similar human methods now being used [40]. More detailed discussion about these tasks and their associated psychopharmacology are reviewed in [1,18,38,40].

## New developments in methods to study reward-related cognition and affective biases

Advances in measuring reward-related behaviour in humans, such as the move away from subjective, questionnaire-based methods and the development of computer-based neuropsychological tasks, have supported reverse translation into animal research. An excellent example of this is the probabilistic reward learning task [41, 42, 43] and probabilistic reversal learning task [44,45]. In both models, the animal is required to learn contingencies associated with different cues despite receiving false feedback resulting from the probabilistic nature of the reward delivery. Probability of reward can be altered to increase task difficulty, and response bias, discriminability, accuracy, reaction time and sensitivity to positive and negative feedback can all be collected. Tasks using operant chambers with spatial cues or tones, a touchscreen task and methods using odour cues have all been piloted [1,43]. However, it should be noted that there are marked differences between human and rat data in terms of the proportion of lose-shift responses after misleading negative feedback suggesting the underlying biology may be different.

In our own laboratory, we started looking at how affective states could modify reward learning as part of our research into affective biases [46,47]. Affective bias is a term used to describe how affective states influence cognitive processes to bias responding. Biases have been observed across many different cognitive domains with clinical studies demonstrating impairments in emotional interpretation, memory and decision-making, and in response to acute doses of antidepressants [48, 49, 50, 51,52^•^]. To translate this work to animals, we needed to use stimuli and outcomes appropriate to the species [53^••^]. This was achieved by pairing previously neutral cues with emotionally-valenced outcomes: obtaining reward or avoiding punishment. The use of reward-related learning and memory in these tasks, as well as decision-making behaviour, has led to new insights into how these are modified in psychiatric disorders.

The affective bias test (ABT, Figure 1, panel b) was designed to investigate how affective state at the time of learning would impact on the subsequent reward value attributed to the memory of that experience [54]. Animals are first presented with a simple task where they are required to learn the association between a specific cue (a digging substrate) and a rewarding outcome (a food pellet). These Pavlovian associations are leant on independent days with the reward value kept constant, but a manipulation can be applied before one of the cue-reward associations. Because of the within-subject design, a subsequent choice test can be used to assess the individual animal’s preference between the two previously learnt reward-associated cues. Where the manipulation leads to a positive affective state, animals show a preference for the reward-cue paired during treatment versus the cue paired during the control condition, a positive bias [54, 55, 56]. The opposite is seen with manipulations which induce a negative affective state, or are pro-depressant, with animals biasing their responding away from the treatment-paired cue [54,55,57]. Importantly, positive biases are seen with acute antidepressant treatment whilst negative biases are observed following treatments known to have pro-depressant effects (Figure 1, panel b, effect on acquisition and consolidation). Administering treatments immediately post discrimination learning showed similar effects, suggesting consolidation is the most likely aspect of learning being modulated [54]. Interestingly, we did not find any effects with dopaminergic drugs, suggesting a distinct underlying neurobiology [54]. Further studies have linked the modulation of reward-learning in this task to the amygdala [58]. Whilst the amygdala is known to play a role in Pavlovian conditioning, we observe an interesting dissociation between biases induced by manipulations of affective state (attenuated) versus those induced by changes in absolute reward (not affected).

To test whether these treatment-induced biases can be subsequently altered, we can also administer a treatment immediately before the choice test (Figure 1, panel b, effect on recall, [58]). In this version of the ABT, we observe an interesting dissociation between delayed and rapid onset antidepressants [58]. The NMDA antagonist, ketamine but not the monoaminergic antidepressant, venlafaxine, was able to attenuate previously learnt biases which we propose may help explain its rapid onset of action. We linked this effect to the medial prefrontal cortex where inactivation using the GABA agonist muscimol induced similar effects. To date, we have shown that the ABT can provide a novel method to study how manipulations at the time of learning can impact on long term memory for reward and how this influences behaviour when the animal re-encounters the cue which predicts that specific reward.

The assay may also be interesting for research relating to the learning of rewards of different absolute values, an aspect of reward-related cognition which is not currently addressed within the basic research field. We made some early attempts to test this by developing a modified version of the assay which measures associations between distinct, independently learnt cues that predict different values of reward [57]. We have termed this a reward-induced positive bias and normal animals show a bias towards the cue paired with a higher value reward. Initial studies have focussed on putative models of depression and we have observed significant deficits in reward memory when animals are treated with chronic corticosterone, chronic pro-depressant drug treatments and in animals exposed to early life adversity [59^••^]. Importantly, this effect is distinct from consummatory deficits as, with the exception of the chronic corticosterone treatment, the same animals do not show impairments in the SPT [59^••^]. A similar deficit in reward-induced positive bias has also been observed in the sub-chronic PCP model of schizophrenia which similarly does not show impaired reward-related behaviour in other methods [60].

Another task which investigates affective biases, but this time in relation to reward-related decision-making, is the judgement bias task [46,61]. The task tests whether the affective state of an animal biases decisions-making during presentation of ambiguous information. There have been two different versions developed which either use reward versus punishment avoidance or high-reward versus low-reward ([46], see Figure 1, panel c). Animals are first trained to associate specific cues (reference cues) with predicting a high-value or low-value reward (or avoidance of punishment). Once a stable baseline level of responding has been achieved, the animals are presented with intermediate ambiguous cues and their responses (anticipation of high-value or low-value reward) used to indicate a positive or negative bias. Both versions of the task have shown that animals in a putative negative affective state are more likely to anticipate a less positive or negative event [61, 62, 63, 64, 65, 66, 67, 68]. Pharmacological studies have revealed an interesting dissociation between acute and chronic effects of monoaminergic antidepressants, with effects only observed following chronic administration [64]. In contrast, the rapid-onset antidepressant ketamine induces an immediate positive bias [64]. A major advantage of the judgement bias task is its ability to differentiate between biases in decision-making (responses made to ambiguous probe cues) and changes in consummatory or motivational aspects of reward (responses made to reference cues). In our studies, we have been able to demonstrate specific changes in decision-making behaviour without effects on consummatory or motivational measures [63,64].

## Conclusions

Deficits in reward processing are no longer considered a unitary construct and these new developments in animal models support this. A patient may report a loss of interest in pleasurable activities, but the underlying cause of this deficit may lie within one or more of several distinct domains which ultimately contribute to reward-related behaviour. Questionnaire measures are limited in terms of their ability to differentiate between these different subcomponents. To address this, new translational methods to study reward-related cognition in both human and animal studies have been developed and are starting to yield important findings. Further studies comparing different patient populations using a test battery designed to probe different types of reward deficit are needed. Similarly, more detailed validation of the rodent tasks discussed here are required, particularly to extend the findings from depression-related research to other areas including other psychiatric disorders and neurodegenerative conditions. On the basis of clinical findings, impairments in reward-related cognition may represent a more relevant phenotype and the new tasks discussed here offer a pre-clinical approach to study these.

## Conflict of interest statement

The authors have no conflicts of interest to declare. ESJR has current research funding from Boehringer Ingelheim and Eli Lilly which supports research in this area but they have had no influence on the content of this article. ESJR has also previously received funding from Pfizer and MSD in the form of academic research grants.

## References and recommended reading

Papers of particular interest, published within the period of review, have been highlighted as

• of special interest
•• of outstanding interest
